# Auer Rod–Like Inclusions in Myeloma Plasma Cells With Aberrant CD34, CD117, and CD33 Expression

**DOI:** 10.1002/jha2.70412

**Published:** 2026-09-24

**Authors:** Olivier Pouliot, Geneviève Payette, Antoine Caillon

**Affiliations:** ^1^ Department of Onco‐Hematology Centre Hospitalier Universitaire de Montreal (CHUM) Montreal Quebec Canada; ^2^ Faculty of Medicine University of Montreal Montreal Quebec Canada; ^3^ Pierre‐Le Gardeur Hospital Terrebonne Quebec Canada; ^4^ Research Center of CHUM Montréal Quebec Canada

## 1 Clinical Presentation

A 73‐year‐old man presented with acute kidney injury and elevated ferritin (1620 ng/mL). Laboratory investigations showed markedly elevated serum κ free light chains (8948 mg/L; κ/λ ratio, 1420), with otherwise normal blood counts, calcium, and serum protein electrophoresis. Imaging demonstrated no lytic bone lesions.

## 2 Morphology and Immunophenotype

Bone marrow aspiration revealed 43% plasma cells with plasmablastic morphology. Rare plasma cells contained needle‐shaped azurophilic cytoplasmic inclusions resembling Auer rods, including one cell with a bundled, “faggot‐cell–like” arrangement (left; Wright–Giemsa stain, ×1000).

**FIGURE 1:**
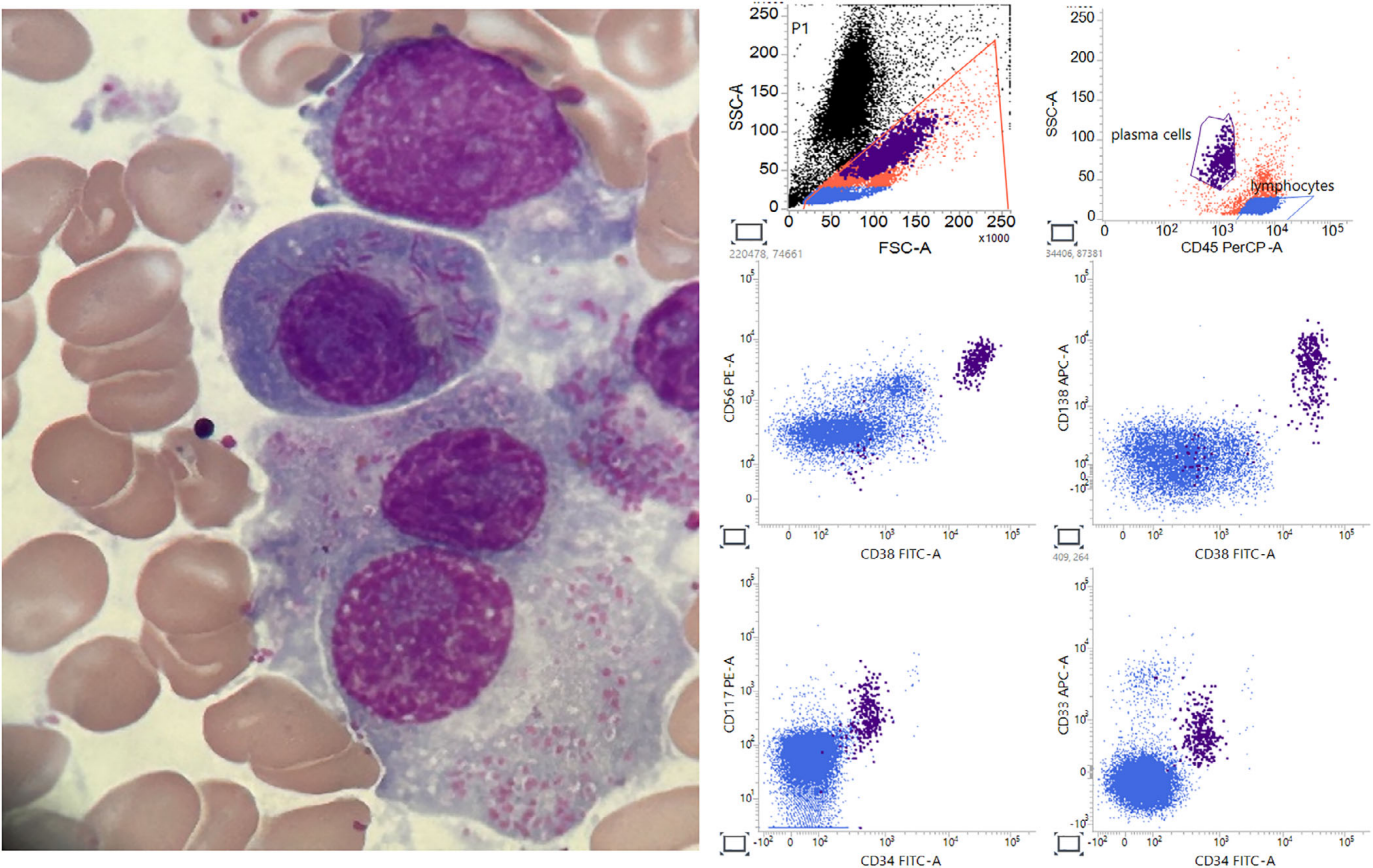
Auer rod–like inclusions in myeloma plasma cells with aberrant myeloid‐associated antigen expression. Bone marrow aspirate smears show plasmablastic plasma cells containing azurophilic Auer rod–like cytoplasmic inclusions (left). Flow cytometry identifies the plasma‐cell population through CD38, CD138, and CD56 expression and demonstrates aberrant expression of CD34, CD117, and CD33 (right).

Flow cytometry identified a monotypic plasma‐cell population with high forward and side scatter. The cells expressed CD38, CD138, and CD56, confirming plasma‐cell lineage. Low‐level expression of CD34, CD117, and CD33 was also observed (right), producing an unusual myeloblast‐like immunophenotype. Flow‐cytometric demonstration of aberrant antigen expression and light‐chain restriction is valuable for identifying neoplastic plasma cells and resolving diagnostically unusual cases (Figure 1) [1].

Auer rod–like inclusions have rarely been described in plasma‐cell neoplasms and have most often been reported in κ‐restricted myeloma [2, 3]. Unlike true Auer rods, these structures have demonstrated lysosomal enzyme activity and lack the myeloperoxidase‐containing primary‐granule material characteristic of myeloid Auer rods [2, 3]. Their occurrence in non‐myeloid cells may therefore create a diagnostic pitfall, particularly when a concomitant myeloid neoplasm is suspected [4].

In this case, the coexistence of Auer rod–like inclusions with CD34, CD117, and CD33 expression produced an unusual morphological and immunophenotypic resemblance to myeloid blasts. Correlation with plasma‐cell markers and light‐chain restriction was essential to establish the correct lineage.

## 3 Key Learning Points

- Auer rod–like inclusions can occur rarely in plasma‐cell neoplasms.
- These inclusions do not establish myeloid lineage.
- Aberrant CD34, CD117, and CD33 expression may accentuate resemblance to myeloblasts.
- Integrated morphological and flow‐cytometric assessment can prevent misclassification as a myeloid malignancy.

## 4 Conclusion

Recognition of this rare morphological–immunophenotypic overlap is important for distinguishing plasmablastic myeloma from an acute myeloid neoplasm.

## Author Contributions

G.P. established the diagnosis. O.P. collected and interpreted the clinical, morphological, and immunophenotypic data and drafted the manuscript. A.C. conceptualized and supervised the report, interpreted the flow‐cytometric findings, and critically revised the manuscript. All authors approved the final version and accept accountability for the work.

## Funding

The authors have nothing to report.

## Ethics Statement

According to institutional policy, ethics committee approval is not required for the publication of a single anonymized case report. The report was prepared in accordance with institutional requirements and the principles of the Declaration of Helsinki.

## Conflicts of Interest

The authors declare no conflicts of interest.

## Data Availability

The data supporting this report are available from the corresponding author upon reasonable request, subject to institutional requirements and the protection of patient confidentiality.
